# Plasma Neurofilament Light Chain in Patients Affected by Alzheimer’s Disease with Different Rate of Progression: A Retrospective Study on an ADNI Cohort

**DOI:** 10.3390/brainsci15090924

**Published:** 2025-08-27

**Authors:** Giuseppe Virga, Bruno Di Marco, Valeria Blandino, Tommaso Piccoli

**Affiliations:** 1 Department of Biomedicine, Neurosciences, and Advanced Diagnostics (Bi.N.D.), University of Palermo, 90129 Palermo, Italy; giuseppe.virga13@gmail.com (G.V.); bbrunodimarco@gmail.com (B.D.M.); 2 Cognitive and Memory Disorders Clinic, AOUP “Paolo Giaccone” University Teaching Hospital, Department of Biomedicine, Neurosciences, and Advanced Diagnostics (Bi.N.D.), University of Palermo, 90129 Palermo, Italy; valeriablandino0@gmail.com

**Keywords:** Alzheimer’s disease, dementia, biomarkers, plasma neurofilament, progression

## Abstract

**Background:** Alzheimer’s disease (AD) shows highly variable progression rates among individuals. Plasma neurofilament light chain (NfL) has emerged as a potential biomarker of neurodegeneration. **Objectives:** this study aimed to evaluate the predictive value of plasma NfL in estimating the rate of clinical progression (RoP) in AD. **Methods:** we retrospectively analyzed 87 AD patients from the Alzheimer’s Disease Neuroimaging Initiative (ADNI) database. We stratified patients into two groups based on the median RoP, which was calculated from longitudinal Mini-Mental State Examination (MMSE) score evaluations: slow decliners (SD) and fast decliners (FD). We then compared plasma NfL levels between the two groups and examined their relationship with the progression rate. **Results:** patients with faster decline rates had higher levels of NfL. Logistic regression (LR) analysis revealed a strong correlation between plasma NfL levels and disease progression rates. Furthermore, a multivariate model incorporating Aβ42 levels improved predictive accuracy. **Conclusions:** these findings suggest that plasma NfL could serve as a valuable biomarker for monitoring the progression of Alzheimer’s disease, identifying patients at greater risk of rapid decline, and optimizing therapeutic strategies and clinical management. Future studies on larger cohorts will be essential to confirm and further explore these observations.

## 1. Introduction

AD is the most common form of dementia and one of the leading causes of disability in the elderly population. It is a progressive neurodegenerative disorder of the central nervous system (CNS), characterized by a gradual and irreversible decline in cognitive functions, including memory, language, executive functions, and spatial orientation [1]. From a neuropathological perspective, AD is marked by the extracellular deposition of β-amyloid (Aβ) in the brain parenchyma and within cerebral vessels, the intraneuronal accumulation of hyperphosphorylated tau into neurofibrillary tangles, and widespread synaptic dysfunction and neuronal loss [2,3]. These alterations begin years, or even decades, before the appearance of overt clinical symptoms, making early disease detection and the identification of prognostic biomarkers capable of capturing the pathological processes before irreversible damage occurs critically important [4].

Traditionally, the clinical diagnosis of AD relied on the evaluation of cognitive and functional abilities through standardized tools such as the MMSE and functional scales for daily living activities [5]. However, this approach lacked specificity and typically allowed the disease to be identified only at symptomatic stages. In 2011, the National Institute on Aging and the Alzheimer’s Association (NIA-AA) revised the diagnostic criteria to include the use of biomarkers, thereby opening the possibility of earlier and more accurate diagnosis, particularly in the prodromal stage of the disease (mild cognitive impairment, MCI) [6,7]. This change was supported by growing evidence that the pathological mechanisms of AD remain silent for a long period and can be detected through biomarkers found in cerebrospinal fluid (CSF), cerebral cortex, and blood [8,9].

The most recent conceptualization of AD, as expressed by the A/T/N framework, defines the disease based on the presence or absence of three classes of biomarkers: amyloid pathology (A), tau pathology (T), and neurodegeneration (N) [10,11]. This biological approach has improved diagnostic precision and enabled more refined stratification of patients based on underlying pathophysiological mechanisms [12]. However, one of the unresolved clinical challenges is the considerable inter-subject variability in disease progression: while some patients remain stable for years, others experience rapid cognitive and functional decline. This heterogeneity is influenced by both intrinsic and extrinsic factors, such as age, sex, genetic predisposition (e.g., APOE ε4), comorbidities, environmental factors, and lifestyle [13,14].

Predicting the RoP is a priority for both clinical management and the targeted inclusion of patients in clinical trials. The first models aimed at forecasting RoP were based on cognitive variables (e.g., changes in MMSE scores) and diagnostic delay, defined as the interval between symptom onset and clinical evaluation, as proposed by Doody and colleagues in the concept of the “pre-progression rate” [15]. However, estimating the precise onset of disease is often subjective and prone to bias, particularly when based on reports from patients’ family members. In the last few years, research has shifted toward combining clinical knowledge with machine learning algorithms and multivariate models to achieve more objective and accurate progression predictions [16,17].

In parallel, the field of biomarker research has expanded, especially regarding those associated with synaptic dysfunction and axonal damage. Neurofilaments (Nfs) have garnered increasing interest as robust markers of axonal degeneration [18]. These neuron-specific cytoskeletal proteins, composed of three subunits (light, medium, and heavy), play a key role in axonal stability and axoplasmic transport. Under physiological conditions, their turnover is very low, but in the presence of neurodegeneration, they are released in large amounts into the extracellular space, eventually diffusing into the CSF and, subsequently, into the blood. Among the isoforms, the light chain (NfL) is the most abundant, soluble, and easily measurable in biological fluids [19,20]. Its concentration has been investigated across numerous neurological disorders, including AD, frontotemporal dementia (FTD), amyotrophic lateral sclerosis (ALS), multiple sclerosis (MS), atypical parkinsonian disorders (APD), and traumatic brain injury (TBI), demonstrating its role as a general, although non-specific, marker of axonal damage [18,21].

In AD, both CSF and plasma levels of NfL are elevated even in early stages and correlate with clinical severity and disease progression rates [22,23,24]. Higher NfL concentrations in CSF have been associated with an increased risk of developing MCI in cognitively healthy individuals, faster cognitive decline in MCI patients, and lower overall survival in individuals with AD dementia [25,26,27]. In familial forms of AD, plasma NfL levels have been shown to increase as early as 16 years prior to the expected onset of symptoms in mutation carriers, suggesting its role as an early biomarker of disease conversion [28]. Furthermore, plasma NfL has been proven to be useful in predicting clinical progression and conversion from MCI to dementia, with significantly lower concentrations observed in stable MCI patients compared to those progressing to AD [23,28,29]. From a technical standpoint, measuring plasma NfL offers a non-invasive, reproducible, and well-suited method for longitudinal studies. Ultra-sensitive technologies, such as Single-Molecule Array (SiMoA), enable the detection of NfL even at very low concentrations, paving the way for its implementation in large-scale clinical use [30].

Despite strong evidence supporting the role of NfL as a marker of neurodegeneration, few studies have directly investigated its ability to predict the rate of cognitive decline in biologically confirmed AD patients. Moreover, while classical CSF biomarkers (Aβ42, tTau, and pTau) provide diagnostic information about amyloid and tau pathology, their prognostic value remains debated [9]. This underlines the need for comparative analyses evaluating both established and emerging biomarkers in relation to RoP. In particular, increasing value is being placed on the plasma-based assessment of biomarkers, and it is within this context that the present study is situated.

Phenotypic heterogeneity and variable disease progression are major obstacles in developing effective therapies. To overcome these limitations, there is growing interest in integrated prognostic models combining multiple biomarkers, representing amyloid load, tau pathology, neurodegeneration, and synaptic dysfunction, thus offering a more comprehensive view of the disease and promoting a personalized medicine approach [10].

In this context, the present study aims to evaluate the prognostic value of CSF biomarkers (Aβ42, tTau, pTau) and emerging biomarkers, particularly plasma NfL, in predicting disease progression rate in patients with AD. Using a well-characterized cohort from the ADNI database, we analyzed the association between baseline biomarker levels and longitudinal cognitive decline, measured by MMSE score evaluations. The objective is to determine whether plasma NfL, alone or in combination with other markers, can serve as a reliable marker of disease aggressiveness and support early stratification of patients for targeted therapies and clinical trials.

## 2. Materials and Methods

### 2.1. Study Population

Data for this retrospective observational study were obtained from the ADNI database (http://adni.loni.usc.edu/ (accessed on 13 February 2024)).

The ADNI project aims to develop and validate biochemical and neuroimaging biomarkers for the detection, monitoring, and treatment of AD. All participants, or their authorized representatives, provided written informed consent, which was approved by the Institutional Review Boards (IRBs) of the participating ADNI centers. A detailed description of the inclusion criteria is available on the ADNI website (www.adni-info.org (accessed on 13 February 2024)).

The ADNI 1 database subset considered for this study included a total of 267 patients, which was granted by Blennow K, MD, PhD from Clinical Neurochemistry Laboratory, Dept. of Neuroscience and Physiology, University of Gothenburg, and submitted on 20 September 2016.

A total of 87 participants with a baseline diagnosis of AD dementia were included.

These participants were selected from an initial ADNI 1 cohort of 267 individuals based on predefined inclusion and exclusion criteria. Inclusion criteria were as follows: clinical or biological diagnosis of AD; availability of CSF biomarkers (Aβ42, tTau, and pTau) measured at the time of diagnosis (t_0_); and at least two MMSE assessments, one at t_0_ and one at 12 months (t_1_) or 24 months (t_2_). Exclusion criteria were as follows: negative or uncertain AD diagnosis; insufficient MMSE evaluations at t_0_ and 12-month follow-up (t_1_); absence of plasma NfL measurement; availability of follow-up MMSE or NfL data without baseline (t_0_) measurements; missing measurement of at least one CSF biomarker (Aβ42, tTau, or pTau) at t_0_; and non-adherence to the follow-up protocol.

Eligible individuals had available baseline CSF and plasma biomarker data, as well as longitudinal MMSE scores over a follow-up period of at least 12 months. All patients underwent a comprehensive clinical and neurological evaluation, brain magnetic resonance imaging (MRI), fluorodeoxyglucose positron emission tomography (FDG-PET), CSF collection, and venous blood sampling as part of the standardized diagnostic protocol. APOE genotyping was also performed (Applied Biosystems, Foster City, CA, USA). For inclusion in the present analysis, participants were required to have at least two MMSE evaluations within a 12–24-month time window. The details of demographic and clinical features of participants are reported in Table S1.

The rate of cognitive decline, or RoP, was calculated using the formula proposed by Doody et al. [15]:

$$RoP=\;\frac{First\;MMSE-Last\;MMSE}{time\;of\;follow\;up\;(months)}$$

Subsequently, participants were stratified into two groups, SD and FD, based on the median RoP value, following the approach described by Blandino and colleagues [27].

### 2.2. Analyses of CSF and Plasma Samples

Within the ADNI framework, standardized procedures were implemented to ensure consistency and reliability of the collected data. All analyses were conducted in certified laboratories following strict quality control and assurance protocols (https://adni.loni.usc.edu/help-faqs/adni-documentation/ (accessed on 13 February 2024)).

CSF samples were collected via lumbar puncture from fasting participants, typically between 8:00 and 10:00 a.m. The first 2 mL of CSF were discarded to minimize potential blood contamination, and the subsequent 20 mL were collected for biomarker analysis. Following collection, CSF was processed rapidly to preserve biomarker integrity. The first 3 mL were used for standard analyses, including cell count, glucose, and total protein measurement, performed in local laboratories. The remaining CSF was centrifuged and aliquoted into polypropylene tubes, then immediately frozen and stored at −80 °C. Finally, frozen aliquots were shipped on dry ice to the central laboratory for specialized biomarker assessment. CSF biomarker concentrations were measured using highly sensitive and specific immunoassay techniques. Aβ42, t-Tau, and p-Tau181 levels were determined using chemiluminescence enzyme immunoassay (CLEIA) on the Lumipulse G System (Fujirebio, Ghent, Belgium).

For the quantification of plasma NfL levels, ADNI employed SiMoA technology (Quanterix, Billerica, MA, USA). Blood samples were collected from fasting participants between 8:00 and 10:00 a.m. The analysis utilized a combination of monoclonal antibodies specifically targeting NfL, with purified bovine NfL used as the calibrator. Each sample was analyzed in singlicate to determine the absolute concentration of plasma NfL.

All samples were measured in duplicate, except for one (due to technical reasons). Analytical sensitivity was <1.0 pg/mL, and no sample contained NfL levels in plasma below the limit of detection (LOD).

### 2.3. Statistical Analyses

Statistical analyses were conducted using Python programming language (version 3.9) within Jupyter Notebook (version 6.5.1) environment. Data management and manipulation were performed using Pandas library, while numerical computations and statistical operations were carried out with NumPy. Inferential statistical analyses were conducted with SciPy, which allowed for the application of Spearman’s correlation test, the Mann–Whitney U test for group comparisons, and the chi-square test for categorical variables. LR analyses were performed using the Statsmodels package. The diagnostic performance of biomarkers was assessed through Scikit-learn library, which enabled the construction of ROC curves and the calculation of key performance metrics, including the area under the curve (AUC), sensitivity, and specificity. Data visualization was carried out using Matplotlib (version 3.6.3) and Seaborn (version 0.11.2).

Normality was assessed using the Shapiro–Wilk test. Normally distributed continuous variables were reported as mean ± standard deviation (SD), while skewed data were presented as median and interquartile range (IQR). Categorical variables were reported as frequencies. Variables with normal distribution were analyzed using parametric tests (Student’s *t*-test, Pearson correlation), whereas non-normally distributed variables were assessed with non-parametric methods, including the Mann–Whitney U test for group comparisons and Spearman’s rank correlation coefficient for association analyses. No correction for multiple comparisons was applied, given the exploratory nature of this study and the limited sample size. This approach may increase the risk of error, and findings should therefore be interpreted with caution.

The Receiver Operating Characteristic (ROC) curve analysis was employed to evaluate the discriminative ability of selected biomarkers in distinguishing patients grouped by the median value of their RoP. For each biomarker, the optimal threshold (cut-off), sensitivity, specificity, AUC with 95% confidence interval (CI), and the likelihood ratio (LR) were calculated. AUC values were interpreted as follows: “excellent” (AUC 0.90–1.00), “good” (AUC 0.80–0.89), “fair” (AUC 0.70–0.79), “poor” (AUC 0.60–0.69), and “fail” (AUC 0.50–0.59), indicating no discriminative ability.

In parallel, LR analyses were conducted to estimate the predictive power of the biomarkers and the RoP, using a binary outcome based on whether RoP values were above the sample median. Additionally, predictive models were developed by combining multiple biological variables, with model selection guided by the Akaike Information Criterion (AIC) and the Bayesian Information Criterion (BIC), to identify the most parsimonious and effective configurations. A *p*-value < 0.05 was considered statistically significant. Variable inclusion was guided by biological plausibility and prior evidence, and highly collinear predictors were not entered simultaneously in the same model. Given the limited sample size, these models should be interpreted with caution, as they may be exposed to overfitting.

## 3. Results

### 3.1. Demographic and Clinical Features of Participants

We conducted a retrospective observational study involving 87 patients diagnosed with AD (*n* = 87), with the aim of investigating whether the RoP could be predicted based on CSF biomarker levels and plasma NfL concentrations measured at the time of diagnosis. For this purpose, patients were stratified into two groups according to the median RoP value: a slow progression group (SD: RoP < 0.1$\bar{6}$) and a fast progression group (FD: RoP > 0.1$\bar{6}$). As shown in Table 1, no statistically significant differences were found between the two groups in terms of clinical and demographic characteristics.

To assess the association between RoP and demographic or clinical variables, Spearman’s rank correlation analyses were performed. These analyses did not reveal any statistically significant correlations, as reported in Table S2.

### 3.2. CSF and Plasma Biomarkers in AD Patients Stratified on the Basis of Their RoP

Biomarker levels for the entire study population are reported in Table S3. The relationship between RoP scores and CSF biomarkers was investigated using Spearman’s rank correlation analysis. Significant correlations with RoP were found only for plasma NfL levels (*p* < 0.001) (Table 2).

When stratifying AD patients into the two previously described groups, we observed that those in the FD group exhibited higher plasma NfL concentrations compared to the SD group, while other biomarkers did not reach statistical significance (Table 3). Aβ42 narrowly missed statistical significance (*p* = 0.0507).

**Figure 1:**
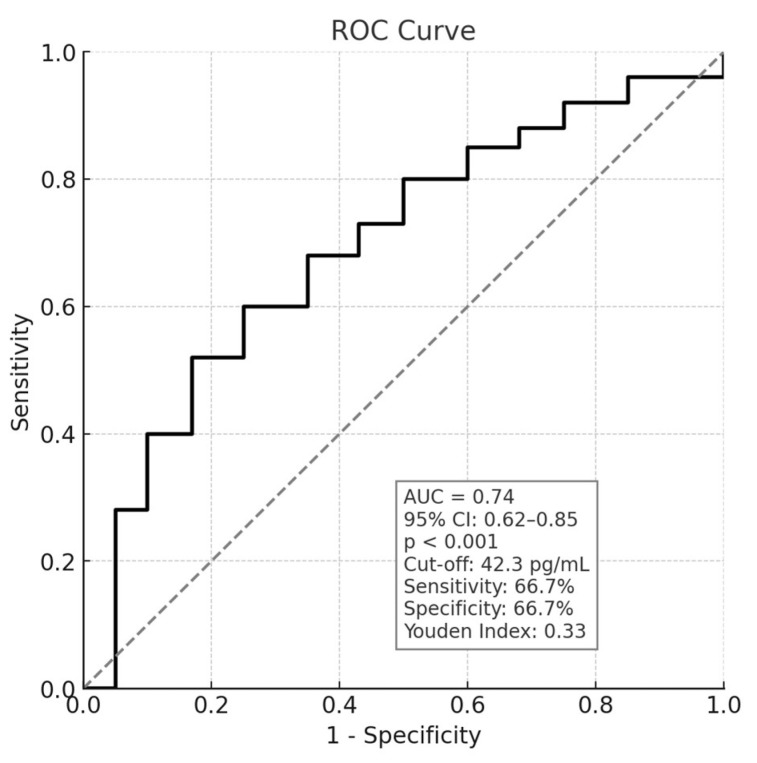
ROC curve analysis of Plasma NfL, with cut-off value, sensitivity, and specificity, calculated according to Youden’s index. The solid line represents the ROC curve, showing the relationship between sensitivity and 1-specificity for different cut-off values. The dashed diagonal line represents the reference line of random classification (no discriminative ability).

Subsequently, we assessed the diagnostic accuracy of plasma NfL levels in discriminating SD from FD patients. ROC curve analysis demonstrated a significant discriminative ability of NfL to differentiate FD from SD patients (AUC = 0.74, 95% CI: 0.62–0.85, *p* < 0.001). The optimal cut-off for plasma NfL, determined using Youden’s index, was 42.3 pg/mL, yielding a sensitivity of 66.7% and a specificity of 66.7% (Figure 1).

### 3.3. Predictive Ability of Biomarkers and Confounding Variables for RoP in AD Patients

In exploring the contribution of demographic and clinical characteristics to predict the RoP in AD patients, we did not identify any significant role for these factors as potential confounders (Table S4).

We subsequently analyzed the predictive value of CSF biomarkers and plasma NfL levels. To this end, we used pathological cut-off values to stratify our population: <650 pg/mL for Aβ42, >416 pg/mL for tTau, and >61 pg/mL for pTau, as previously proposed by Agnello et al. [31]. For plasma NfL, we adopted the previously established cut-off of 42.3 pg/mL. We found that only plasma NfL levels above the cut-off were associated with an increased risk of faster progression (*p* = 0.0023) (Table 4).

We then performed multivariate LR analyses to assess whether combining NfL with other CSF biomarkers, APOE ε4 genotype, and MMSE scores would improve the prediction of RoP in our population. To this aim, we fitted several predictive models and compared them using AIC and BIC criteria to identify the best-fitting model. NfL was selected as the “base variable” in our models, as it showed the highest statistical significance in the univariate LR. As shown in Table 5, the intercept values of all models reached statistical significance, with the exception of Model 5 (base + MMSE). Excluding the base model (AIC = 92.69; BIC = 97.62), Model 1, which combines NfL with Aβ42, demonstrated the best fit compared to the other tested models.

**Figure 2:**
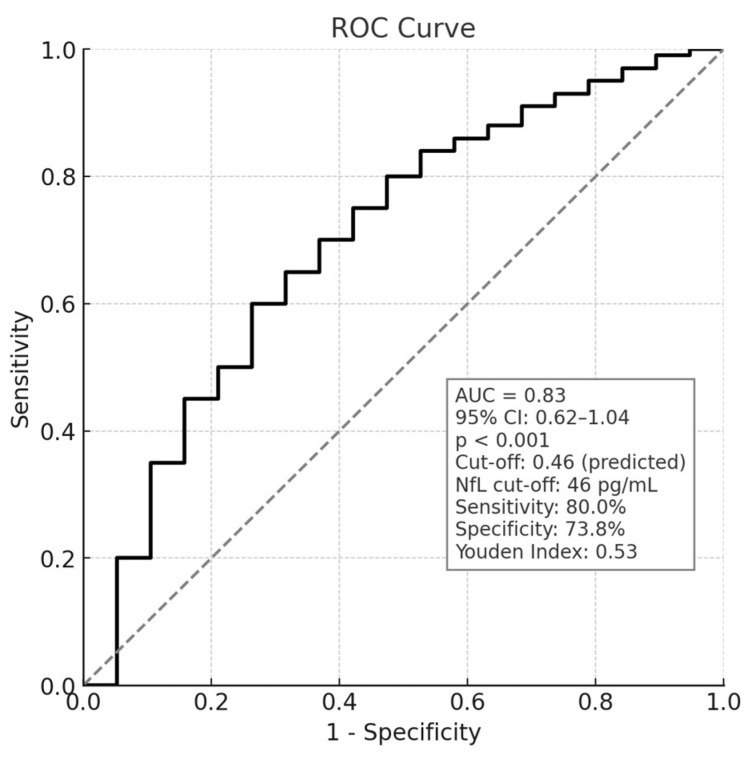
ROC curve analysis of the LR model based on plasma NfL and Aβ42, with cut-off values, sensitivity, and specificity calculated according to the Youden Index. The solid line represents the ROC curve, showing the relationship between sensitivity and 1-specificity for different cut-off values. The dashed diagonal line represents the reference line of random classification (no discriminative ability).

To assess the discriminative capacity of the predictive model combining plasma NfL and Aβ42, a ROC curve was generated. The AUC was 0.83 (95% CI: 0.62–1.04), with a significant *p*-value (*p* = 0.001). The Youden index identified an optimal cut-off probability of 0.46, which represents the threshold beyond which patients are classified as belonging to the fast-declining (FD) group. At this threshold, the model’s sensitivity was 80.0% and specificity was 73.8%, indicating a good balance between identifying true FD cases and minimizing false positives (Figure 2, Table S5).

## 4. Discussion

One of the most challenging aspects in the study of neurodegenerative diseases is the ability to predict their course, particularly the rate at which cognitive and functional decline progresses. The RoP represents a clinically useful measure for describing the trajectory of decline over time, reflecting the gradual loss of neurons and the progressive disruption of cerebral networks [32]. This parameter, initially described by Doody et al. [33], has proven informative in characterizing the progression of AD, even during its early stages of the disease. Numerous factors, such as age at onset, baseline severity, genetic profile, comorbidities, and environmental influences, have been proposed as potential determinants of RoP, although their actual predictive capacity remains debated [34]. In this context, the use of biomarkers has emerged as a promising tool for predicting the speed of disease progression across several neurodegenerative disorders, including AD [35].

In light of these considerations, we conducted a retrospective observational study to explore the association between available plasma and CSF biomarkers at baseline and the RoP, regardless of the patients’ clinical stage.

In our sample, no significant correlations were found between RoP and demographic or clinical characteristics, nor with the APOE genotype, suggesting that such variables, at least in the studied population, do not have predictive value [36,37,38]. In contrast, biomarker analysis revealed a noteworthy finding: a statistically significant positive correlation between RoP and plasma NfL levels, while CSF biomarkers (Aβ42, t-Tau, and p-Tau) showed no significant associations. These results align with an increasingly robust body of evidence supporting plasma NfL as a reliable indicator of active neuroaxonal damage [39]. When stratifying patients according to the median RoP value, only plasma NfL levels were significantly different between groups, with higher concentrations observed in those with faster progression. Although the difference in Aβ42 levels between SD and FD did not reach statistical significance in the univariate comparison (*p* = 0.0507), this near-threshold value may indicate a trend toward lower concentrations in FD patients. Given that reduced CSF Aβ42 reflects greater amyloid burden, which has been linked to faster cognitive decline in AD [40], this finding could be clinically relevant. Confirmation in larger, independent cohorts will be needed to establish whether this trend represents a true biological signal. Furthermore, LR analysis confirmed that baseline plasma NfL was the only significant predictor of a faster RoP. The identification of an optimal cut-off yielded satisfactory performance in terms of sensitivity and specificity, outlining a potentially useful clinical threshold to identify individuals at higher risk of rapid cognitive decline. If validated in larger cohorts, this cut-off could serve as an early prognostic indicator in routine clinical practice. Additionally, we explored a multivariate model that included both plasma NfL and Aβ42 levels. Although Aβ42 alone did not show independent significance in the univariate analysis, its integration into the model improved overall discriminative accuracy. This finding suggests that the combined use of multiple biological markers, even if individually weak, may provide a more robust and clinically relevant predictive value compared to the use of NfL alone. Such multivariate approaches reflect a shift toward personalized risk models aligned with the principles of precision medicine, aiming to predict disease trajectory and inform tailored clinical decision-making [41].

The present study corroborates previous findings on the prognostic role of NfL in the CSF, as reported by Blandino et al. [27], and extends these observations to plasma, a fluid that is more accessible and less invasive to obtain. These results are consistent with prior studies proposing NfL as a systemic biomarker of neurodegeneration [39,42,43]. Several studies have demonstrated the predictive value of NfL in various neurological disorders, supporting its role as a cross-disease prognostic indicator [25,44,45]. Elevated NfL levels have been associated with faster disease progression or reduced survival in conditions such as ALS [46,47], Parkinson’s disease (PD) [48], MS [49], frontotemporal lobar degeneration (FTLD) [50], and stroke [18,21]. These findings further underscore the relevance of NfL as a sensitive marker of active neurodegeneration. Our findings are consistent with a growing body of evidence supporting plasma NfL as a biomarker of neurodegeneration. Prior studies have demonstrated its value across different clinical and biological contexts, including the characterization of NfL trajectories in autosomal dominant AD [51], systematic reviews and meta-analyses of its diagnostic utility [52], and longitudinal studies in subjective cognitive decline and MCI [29,53]. By comparison, the present work extends these observations by focusing on the RoP within a clinically diagnosed AD cohort, thereby providing complementary insights into its prognostic role. It is important to note that while the cited works represent landmark contributions, our study differs substantially in aims, methodology, and clinical implications. For example, investigations on autosomal dominant AD provide crucial mechanistic insights but are not directly generalizable to the broader population of sporadic AD, which represents most clinical cases. Similarly, meta-analyses emphasize diagnostic accuracy across heterogeneous populations but do not address prognosis. Studies in SCD and MCI focus on conversion risk at prodromal stages, whereas our analysis targets patients with established AD, highlighting the heterogeneity of progression and the ability of plasma NfL to distinguish fast- from slow-progressing individuals. This approach has direct clinical implications, informing prognoses, supporting therapeutic decision-making, and offering a potential tool for stratification in clinical trials. Furthermore, NfL are now formally recognized as clinical progression markers in the latest guidelines by Jack et al., 2024 [11], further strengthening the translational significance of our findings. In this sense, our work provides complementary evidence that moves beyond diagnostic considerations and emphasizes the prognostic value of plasma NfL in the clinical setting. Moreover, NfL has been shown to distinguish not only progression from MCI to dementia [54] but also the early transition from the asymptomatic phase to clinically manifest AD in individuals with familial forms of the disease [55]. In the context of AD, the findings of our study are biologically plausible. AD is characterized by progressive neuronal loss in brain regions critical for memory and cognition, such as the hippocampus and cerebral cortex. In this scenario, neurofilaments, particularly the light chain subunit (NfL), represent a direct marker of neuronal damage [18].

Several pathological mechanisms may contribute to the extracellular release of neurofilaments. These include axonal degeneration triggered by Aβ oligomer and p-Tau deposition in brain tissue; oxidative stress and mitochondrial dysfunction promoting the generation of reactive oxygen species (ROS); and microglial activation with subsequent release of inflammatory cytokines, proteases, and additional ROS [2]. Together, these processes compromise neuronal integrity and lead to NfL release into the extracellular space, where it can be detected in both CSF and plasma. Our findings fit within this framework, emphasizing that plasma NfL reflects the current neurodegenerative burden and may provide useful insights not only for diagnosis but also for prognosis and disease monitoring [28,43]. In line with recent evidence, plasma NfL appears to be a valuable marker of AD progression, while blood-based pTau181 and pTau217 have also shown strong diagnostic and prognostic performance, as recently demonstrated by several studies [56,57].

As with all observational studies, this work presents several methodological limitations that must be considered when interpreting the results. First, the relatively small sample size (n = 87) may limit the generalizability of the findings and increase the risk of statistical error. However, our analytical approach considered not only statistical significance but also effect size estimation, providing a more robust measure of the strength of observed associations regardless of sample size. For example, the difference in plasma NfL levels between groups stratified by RoP revealed a moderate-to-large effect size, supporting the clinical relevance of the observation. Additionally, the use of LR allowed us to control for potential confounding variables and extract meaningful results even with a limited number of subjects. A limitation concerns the risk of overfitting associated with the implementation of multivariate LR models, including more than three predictors in a relatively small sample. Although variable inclusion was guided by biological plausibility and prior evidence, and collinear predictors were avoided, the absence of internal validation procedures further increases this risk. As such, the results of these models should be considered exploratory and require confirmation in larger and independent cohorts. The use of survival models could provide additional insight into the prognostic role of biomarkers. However, the structure of the available ADNI dataset, based on baseline measurements with MMSE follow-up at fixed intervals, did not allow for robust time-to-event analysis, as clinical endpoints such as conversion or survival time were not consistently available for the selected cohort. Nonetheless, it is important to note that the dataset was derived from the ADNI cohort, which may introduce selection bias due to specific characteristics of the participants (including rigorous diagnostic criteria and higher adherence to follow-up), potentially differing from the general AD population. An important limitation of the present study is the absence of detailed clinical information on events that could have influenced plasma NfL levels independently of AD pathology. Conditions such as recent TBI, CNS infections, or other neurological disorders are known to elevate NfL regardless of neurodegenerative processes. Although ADNI applies strict eligibility criteria to minimize the inclusion of participants with significant comorbidities, the lack of granular data on such events in the available dataset prevents us from fully excluding their potential impact. This limitation should be taken into account when interpreting our findings and underscores the importance of integrating comprehensive clinical histories in future studies assessing the prognostic value of NfL. At the same time, it is important to underline that the unknown status of the patients regarding their therapy could have modified the speed of progression of the disease independently of the variations in plasma NfL. Another limitation lies in the cross-sectional nature of the analyzed data, based on a single baseline measurement of biomarkers. In addition, it is important to underline that the use of the median RoP value as a threshold to stratify patients into SD and FD, although methodologically common in exploratory analyses, represents a statistical criterion that may not correspond to a clinically validated cut-off. This approach should therefore be interpreted with caution. Future longitudinal research should aim to establish clinically meaningful RoP thresholds based on robust clinical endpoints, such as conversion to more severe disease stages or accelerated functional decline. Another methodological limitation is that the cut-off values adopted for biomarker classification were derived from previously published studies and were not independently validated within the ADNI cohort [32]. While this choice was made to ensure comparability with prior research and to explore the prognostic value of these thresholds in an independent dataset, it should be emphasized that such values remain exploratory in this context. Future studies should aim to establish and validate cohort-specific cut-offs, ideally based on longitudinal data and clinically meaningful outcomes, before considering their implementation in clinical practice.

The absence of longitudinal data prevents us from evaluating changes in NfL or other markers over time, limiting the development of dynamic predictive models. Repeated measurements would enhance the prognostic utility of these biomarkers, allowing the detection of clinical worsening trajectories linked to specific biological profiles. Furthermore, our findings were not validated in an independent cohort, which represents a critical limitation in confirming the predictive accuracy of plasma NfL. Replication in distinct populations, especially in patients recruited outside the ADNI network, will be essential to strengthen the generalizability of this indicator.

In summary, although the results are promising, future longitudinal studies involving larger and more diverse cohorts should integrate serial biomarker measurements and include external validation analyses to determine whether plasma NfL can be considered a reliable tool to support clinical decision-making in AD management. Based on the current findings and limitations, several directions emerge for further research in this field. A key priority will be to expand the study population by including larger and more heterogeneous cohorts to enhance statistical power and ensure greater clinical representativeness, particularly in comparison to individuals not enrolled in ADNI protocols. Importantly, extending the observation to individuals in the preclinical or prodromal stages of AD will be essential to investigate the predictive capacity of biomarkers, and particularly of the proposed model, from the earliest phases of the neurodegenerative process. Another necessary step is the implementation of longitudinal studies with repeated monitoring of plasma and CSF biomarker levels. This approach would not only improve individual prognostication but also reveal evolution patterns of disease progression, enabling more accurate and dynamic modeling compared to a single baseline snapshot. Simultaneously, incorporating a broader spectrum of biomarkers could further enrich the understanding of the pathological process. Markers related to inflammation, oxidative stress, synaptic damage, or glial dysfunction may help delineate distinct biological subtypes of AD and improve prognostic stratification. In this regard, integrating the assessment of neurogranin as a biomarker of synaptic damage is highly desirable, considering the promising findings from several studies [58,59].

The adoption of detailed clinical profiles and stratified analyses also represents a promising avenue for controlling confounding variables, ensuring greater precision in estimates and statistical modeling. Lastly, an integrated and multimodal approach, combining biological, clinical, genetic, and neuroimaging data, may significantly enhance the predictive accuracy of risk models and provide a more complex and realistic understanding of disease progression. Within this framework, plasma NfL may represent a central node in a network of converging biomarkers with potential applicability in everyday clinical practice.

## 5. Conclusions

This study evaluated the prognostic potential of plasma NfL in Alzheimer’s disease, with a focus on its ability to predict the rate of cognitive decline (RoP). A retrospective analysis was conducted on 87 AD patients, divided into two groups based on disease progression rate. The findings revealed a significant correlation between plasma NfL levels and clinical progression speed, identifying NfL as the only biomarker capable of effectively distinguishing fast from slow decliners. A cut-off value of 46.0 pg/mL, derived from an LR model incorporating Aβ42 data, showed good sensitivity and specificity in patient classification. Despite limitations related to sample size, cross-sectional design, and lack of external validation, the results suggest a potential clinical use of plasma NfL for early patient stratification. Looking ahead, integrating multimodal predictive models with biological biomarkers, clinical data, neuroimaging, and genetic information may enhance diagnostic accuracy and support a personalized approach in AD management. These findings lay the groundwork for future studies aimed at validating plasma NfL as a clinically applicable tool for prognostic stratification in Alzheimer’s disease.

## Figures and Tables

**Table 1:** Demographic and clinical characteristics of participants. Data are expressed as median with IQR. The Mann–Whitney U test was used for continuous variables, while the Chi-square test was used for categorical variables. ^A^ Mini-Mental State Examination at baseline.

Variables	SD (n = 42)	FD (n = 45)	*p*
Age (years)	75.5 (71.1–79.625)	75.7 (68.2–79.6)	0.810
Gender (M/F)	29/13	24/21	1
Follow-up (years)	1.0 (1.0–2.0)	1.0 (1.0–1.0)	0.459
MMSE bl ^A^ (scores)	24 (22–25)	22 (21–25)	0.093
Apo E ε4 (%)	71	67	0.980

**Table 2:** Relationship between RoP scores and participants’ biomarkers analyzed using Spearman’s correlation. Bold values indicate a statistical significance (*p* < 0.05).

Variables	Median Values (IQR)	*rho*	*p*
RoP (*n.a.*)	$0.1\bar{6}\;(0.08\bar{3}\text{–}0.\bar{3}$)	/	/
Aβ42 (pg/mL)	593.6 (455.25–750.2)	−0.089	0.410
tTau (pg/mL)	358.9 (278.75–450.85)	0.083	0.442
pTau (pg/mL)	37.72 (28.13–54.355)	−0.030	0.781
Plasma NfL (pg/mL)	42.3 (32.8–63.05)	0.446	**<0.001**

**Table 3:** Biomarker levels in patients stratified by their RoP. Data are expressed as median and IQR. Differences between groups (SD and FD) were assessed using the Mann–Whitney U test. Bold values indicate statistical significance (*p* < 0.05).

Variables	SD (n = 42)	FD (n = 45)	Effect Size	*p*
Aβ42 (pg/mL)	651.2 (496.575–808.75)	576.4 (447.8–653.9)	0.622	0.0507
tTau (pg/mL)	362.35 (268.4–452.775)	353.7 (286.5–445.4)	0.490	0.875
pTau (pg/mL)	37.97 (27.883–64.228)	37.41 (28.64–47.67)	0.530	0.631
Plasma NfL (pg/mL)	36.9 (30.75–45.975)	61.4 (39.8–90.1)	0.410	**<0.001**

**Table 4:** Binary logistic regression analysis to investigate the predictive role of biomarkers in contributing to RoP scores above the median value (FD > 0.1$\bar{6}$). Bold values indicate statistical significance (*p* < 0.05). ^A^ Standard error.

RoP FD	B	SE ^A^	OD (95%C.I.)	*p*
Plasma NfL (>cut off)	1.386	0.455	4.0 (1.64–9.76)	**0.0023**
Aβ42 (path.)	0.788	0.442	2.2 (0.92–5.23)	0.074
t-Tau (path.)	0.098	0.451	1.1 (0.46–2.68)	0.582
p-Tau (path.)	−0.775	0.534	0.46 (0.16–1.31)	0.256

**Table 5:** Multivariate logistic regression analysis to investigate the contribution of different models in predicting a faster rate of progression (FD > 0.1$\bar{6}$). Bold values indicate statistical significance (*p* < 0.05).

Model	RoP FD	B	SE	*p*	AIC	BIC	LL
Base	NfL > cut-off	−0.735	0.278	**0.008**	92.69	97.62	−44.34
1	base + Aβ42	−1.710	0.540	**0.002**	**87.86**	**95.26**	**−40.93**
2	base + pTau	−0.603	0.311	**0.053**	93.80	101.20	−43.90
3	base + tTau	−0.914	0.357	**0.010**	94.00	101.40	−44.00
4	base + APOE ε4	−0.968	0.479	**0.043**	94.31	101.71	−44.15
5	base + MMSE bl	3.271	3.179	0.304	93.03	100.42	−43.51
6	base + Aβ42 + pTau	−1.577	0.555	**0.004**	88.83	98.69	−40.41
7	base + Aβ42 + tTau	−1.926	0.601	**0.001**	89.11	98.97	−40.56
8	base + Aβ42 + pTau + tTau	−1.833	0.607	**0.003**	89.34	101.67	−39.67

## Data Availability

Data used in this study originate from the Alzheimer’s Disease Neuroimaging Initiative (ADNI) database. Access to ADNI data is granted upon registration and approval through the ADNI Data and Publications Committee at (https://adni.loni.usc.edu/ (accessed on 13 February 2024)); all data are shared without embargo according to ADNI policy.

## Supplementary Materials

The following supporting information can be downloaded at https://www.mdpi.com/article/10.3390/brainsci15090924/s1. Table S1: Demographic and clinical features of all participants; Table S2: Relationship between RoP and demographic or clinical features of participants investigated by Spearman’s correlation analyses; Table S3: Biomarker levels of all participants; Table S4: Logistic regression analysis to investigate the predictive roles of demographic and clinical features of participants in contributing to scores of RoP upper than median value (RoP U-M: >2); Table S5: Confounding matrix and derived metrics with cut-off of plasma NfL + Aβ42 of 0.46.

## Abbreviations

The following abbreviations are used in this manuscript:

A/T/N	Amyloid/Tau/Neurodegeneration
Aβ	Β-Amyloid
AD	Alzheimer’s Disease
ADNI	Alzheimer’s Disease Neuroimaging Initiative
AIC	Akaike Information Criterion
ALS	Amyotrophic Lateral Sclerosis
APD	Atypical Parkinsonian Disorders
AUC	Area Under the Curve
BIC	Bayesian Information Criterion
bl	Baseline
CI	Confidence Interval
CLEIA	Chemiluminescence Enzyme Immunoassay
CNS	Central Nervous System
CSF	Cerebrospinal Fluid
FD	Fast Decliners
FDG-PET	Fluorodeoxyglucose Positron Emission Tomography
FTD	Frontotemporal Dementia
IQR	Interquartile Range
IRB	Institutional Review Board
LR	Logistic Regression
MCI	Mild Cognitive Impairment
MMSE	Mini-Mental State Examination
MRI	Magnetic Resonance Imaging
MS	Multiple Sclerosis
NfL	Neurofilament Light Chain
p-Tau	Phosphorylated Tau
ROC	Receiver Operating Characteristic
RoP	Rate of Progression
SD	Slow Decliners
SE	Standard Error
SiMoA	Single-Molecule Array
t-Tau	Total Tau
TBI	Traumatic Brain Injury
