# Supplementary material for: Plasma Neurofilament Light Chain in Patients Affected by Alzheimer’s Disease with Different Rate of Progression: A Retrospective Study on an ADNI Cohort
Source: Brain Sci. 2025 Aug 27;15(9):924. doi: 10.3390/brainsci15090924 (PMC12468065; doi:10.3390/brainsci15090924)
Supplement: Supplementary file 1 [file brainsci-15-00924-s001.zip › brainsci-3816767-supplementary.pdf]

Supplementary Materials

Article

Plasma Neurofilament Light Chain in Patients Affected by Alzheimer's Disease with Different Rate of Progression: A Retrospective Study on an ADNI Cohort

Table S1. Demographic and clinical features of all participants. Continuous variables are expressed as median with interquartile ranges (IQR), while categorical variables are expressed as relative frequencies. [A] Mini Mental Status Examination at time of diagnosis.

Variables	AD (n=87)
Age (years)	75.5 (70.35 – 79.65)
Gender (% male)	60.9
Follow up (years)	1.0 (1.0 – 2.0)
MMSE bl[A] (scores)	23 (21 – 25)
MMSE 12 months (scores)	21 (18 – 24)
Apo E ε4 (%)	68.9
RoP (*n.a.*)	$0.1\overline{6}$ ($0.08\overline{3}$ – $0.\overline{3}$)

Table S2. Relationship between RoP and demographic or clinical features of participants investigated by Spearman's correlation analyses.

RoP (*n.a.*)	*rho*	*p*
Age (years)	-0.007	0.945
Follow up (years)	0.002	0.985
MMSE bl (scores)	-0.185	0.085

Table S3. Biomarker levels of all participants, data are expressed as median with interquartile range (IQR).

Variables	AD (n = 87)
Aβ42 (pg/ml)	593.6 (455.25 – 750.2)
tTau (pg/ml)	358.9 (278.75 – 450.85)
pTau (pg/ml)	37.72 (28.13 – 54.355)
Plasma NfL (pg/ml)	42.3 (32.8 – 63.05)

Table S4. Logistic regression analysis to investigate the predictive roles of demographic and clinical features of participants in contributing to scores of RoP upper than median value (RoP U-M: >2). Bold font indicates a statistical significance ($p<0.05$).

**RoP FD**	**B**	**SE**	**OD (95%C.I.)**	***p***
Age	-0.003	0.027	1 (0.94 – 1.05)	0.913
Gender	-0.668	0.448	0.51 (0.21 – 1.23)	0.135
Apo E ε4	-0.205	0.295	0.81 (0.46 -1.45)	0.485
MMSE bl	-0.201	0.112	0.82 (0.66 – 1.02)	0.072

Table S5. Confounding matrix and derived metrics with cut-off of plasma NfL + Aβ42 of 0.46.

NfL + Aβ42 : 0.46	**Real: FD**	**Real: SD**
**Predicted: FD**	36	9
**Predicted: SD**	11	31

Sensibility: 80%
Specificity: 73.8%
Positive Predictive Value: 76.6%
Negative Predictive Value: 77.5%
Overall Accuracy: 77%
